# Study of High Levels of Wilms’ Tumor 1 (WT1) Expression in Ovarian Cancers

**DOI:** 10.7759/cureus.104521

**Published:** 2026-03-01

**Authors:** Arshia Fatima, Sunita Pawar, Dnyaneshwar Potpalle, Hari Chandana, Mummareddi Dinesh Eshwar

**Affiliations:** 1 Pathology and Laboratory Medicine, Mahavir Institute of Medical Sciences, Vikarabad, IND; 2 Pediatrics, Mahavir Institute of Medical Sciences, Vikarabad, IND; 3 Pathology and Laboratory Medicine, Dr. Patnam Mahendar Reddy Institute of Medical Sciences, Chevella, IND; 4 General Medicine, Mahavir Institute of Medical Sciences, Vikarabad, IND

**Keywords:** epithelial ovarian tumors, figo staging, immunohistochemistry, serous carcinoma, wilms' tumour 1

## Abstract

Background

Ovarian cancer is a major cause of cancer-related mortality among women and remains a significant diagnostic and therapeutic challenge due to its heterogeneous nature, absence of effective screening strategies, and frequent presentation at advanced stages. Surface epithelial ovarian tumors constitute the largest group of ovarian neoplasms and account for the majority of malignant cases. These tumors encompass multiple histological subtypes with distinct morphological features, biological behavior, and prognostic implications. Accurate histopathological classification supported by immunohistochemical markers is, therefore, essential for diagnosis and prognostication. Wilms’ tumor 1 (WT1) is a zinc finger transcription factor involved in cellular proliferation, differentiation, apoptosis, and regulation of the cell cycle. Although initially described as a tumor suppressor gene, WT1 has been shown to demonstrate oncogenic behavior in several malignancies, particularly ovarian cancer, and is strongly associated with serous ovarian carcinoma.

Methods

This prospective observational study was conducted over a two-year period and included 300 histopathologically confirmed epithelial ovarian tumors. Borderline and malignant epithelial ovarian tumors were included, while benign ovarian tumors, non-epithelial ovarian tumors, inflammatory lesions, and metastatic tumors involving the ovary were excluded. Surgically excised specimens and biopsy samples obtained during routine clinical care were fixed in formalin and processed using standard histopathological techniques. Sections were stained with hematoxylin and eosin for microscopic evaluation and tumor classification. Immunohistochemical staining for WT1 was performed on representative tissue sections, and tumors were categorized as WT1-positive or WT1-negative based on nuclear immunoreactivity. WT1 positivity was analyzed for its association with histological subtype and International Federation of Gynecology and Obstetrics (FIGO) stage.

Results

Malignant tumors constituted the majority of cases, while borderline tumors accounted for a smaller proportion. Serous tumors were the most common histological subtype, followed by mucinous tumors, seromucinous tumors, and poorly differentiated adenocarcinomas. WT1 positivity demonstrated a distinct distribution across tumor subtypes. Nuclear WT1 positivity was predominantly observed in serous carcinomas, while mucinous and seromucinous tumors were WT1-negative. Borderline serous tumors showed WT1 positivity less frequently. WT1 positivity was significantly associated with advanced FIGO stages in serous carcinomas.

Conclusion

WT1 is a sensitive and reliable immunohistochemical marker for serous ovarian carcinoma and is useful in distinguishing serous tumors from other epithelial ovarian neoplasms. The association between WT1 positivity and advanced tumor stage suggests a potential prognostic role. Incorporation of WT1 immunohistochemistry into routine diagnostic practice may enhance tumor classification and provide insights into disease progression in ovarian cancer.

## Introduction

Ovarian cancer remains a leading cause of cancer-related mortality among women and continues to pose a major clinical challenge in gynecologic oncology. Despite its relatively lower incidence compared with cervical and endometrial cancers, ovarian cancer accounts for a disproportionately high number of deaths among gynecological malignancies. This high mortality is largely attributable to late-stage diagnosis in the majority of patients and the intrinsic biological heterogeneity of ovarian tumors, which differ markedly in origin, molecular profile, and clinical behavior [1-4].

The majority of malignant ovarian neoplasms are epithelial in origin and are collectively referred to as epithelial ovarian tumors [2,3]. These tumors represent a biologically diverse group and are histologically classified into several distinct subtypes, including serous, mucinous, endometrioid, clear cell, and seromucinous carcinomas, each characterized by unique morphological features, molecular alterations, clinical behavior, and response to therapy [3,5]. Epithelial ovarian carcinomas are further conceptualized within the dualistic model of ovarian carcinogenesis, which categorizes tumors into type I and type II based on distinct pathogenetic pathways, molecular profiles, and prognostic implications [6]. Accurate histopathological classification is therefore essential, as histological subtype is a key determinant of prognosis, therapeutic decision-making, and patient management [5,6].

In routine diagnostic pathology practice, immunohistochemistry serves as an essential adjunct to conventional histomorphological evaluation, particularly in cases with overlapping, ambiguous, or poorly differentiated microscopic features [7,8]. Among the immunohistochemical markers used in the assessment of ovarian tumors, Wilms’ tumor 1 (WT1) has emerged as a valuable marker in the characterization of epithelial ovarian neoplasms. WT1 is a zinc finger transcription factor involved in the regulation of cellular proliferation, differentiation, apoptosis, and cell cycle control [9]. Although initially identified as a tumor suppressor gene, subsequent studies have demonstrated aberrant WT1 expression and functional involvement in the pathogenesis of several malignancies, including ovarian carcinoma [9,10].

WT1 expression has been shown to be strongly and consistently associated with serous ovarian carcinoma, whereas most non-serous epithelial ovarian tumors, including mucinous, endometrioid, and clear cell carcinomas, typically exhibit absent or minimal WT1 expression [10-12]. This differential expression pattern has established WT1 as a useful diagnostic marker for distinguishing serous carcinoma from other epithelial ovarian neoplasms, particularly in high-grade or poorly differentiated tumors where morphology alone may be insufficient [7,8]. In addition to its diagnostic utility, several studies have suggested an association between WT1 expression and adverse clinicopathological features, including advanced disease stage and unfavorable clinical outcomes, in serous ovarian carcinoma, indicating a potential prognostic role for WT1 [11,12].

Despite the widespread use of WT1 in diagnostic pathology, variations in its expression across different epithelial ovarian tumor subtypes and inconsistencies in its association with disease stage have been reported in the literature [10-12]. Furthermore, data correlating WT1 immunohistochemical expression with International Federation of Gynecology and Obstetrics (FIGO) stage, particularly in borderline tumors and across the full spectrum of epithelial ovarian neoplasia, remain limited and incompletely characterized [10-12].

The present study was therefore undertaken to evaluate the immunohistochemical expression of WT1 in epithelial ovarian tumors and to correlate its expression with histological subtype and FIGO stage. By analyzing WT1 expression patterns in borderline and malignant epithelial ovarian tumors, this study aims to reinforce the diagnostic relevance of WT1 in routine pathology practice and to explore its association with tumor stage within the broader spectrum of epithelial ovarian neoplasia.

## Materials and methods

Study design and setting

The present study was a prospective observational study conducted in the Department of Pathology, MNJ Institute of Oncology and Regional Cancer Centre, Osmania Medical College, Hyderabad, over a period of two years from August 2017 to July 2019. The study was carried out after obtaining approval from the Institutional Ethics Committee (IEC No. ECR/300/Inst/AP/2013/RR-16). Written informed consent was obtained from all patients prior to inclusion in the study. For archived specimens, a waiver of informed consent was granted by the Institutional Ethics Committee.

A total of 300 histopathologically diagnosed epithelial ovarian tumor cases received during the study period were included for analysis.

Study material and selection criteria

The study material comprised surgically excised specimens and biopsy samples of epithelial ovarian tumors. Both borderline and malignant surface epithelial ovarian tumors with adequate tumor tissue for immunohistochemical analysis and complete clinicopathological details, including patient age and histopathological diagnosis, were included in the study.

Benign ovarian tumors, inflammatory lesions of the ovary, metastatic tumors involving the ovary, and non-epithelial ovarian tumors were excluded from the study.

Histopathological processing and evaluation

All surgically excised specimens were fixed in 10% neutral buffered formalin, followed by gross examination and sampling from representative areas of the tumor. The tissues were processed using an automated tissue processor and embedded in paraffin wax. Biopsy specimens were also fixed in 10% neutral buffered formalin and were entirely submitted for tissue processing and paraffin embedding.

From each paraffin block, two sections of 4-5 µm thickness were cut. One section was mounted on an albumin-coated slide for routine hematoxylin and eosin staining, and the other section was mounted on a poly-L-lysine-coated slide for immunohistochemical analysis.

Routine hematoxylin and eosin staining was performed using standard protocols, including deparaffinization, hydration, staining with Harris hematoxylin, differentiation, bluing, eosin counterstaining, dehydration through graded alcohols, clearing in xylene, and mounting with DPX. Histopathological evaluation was carried out, and tumors were classified according to the World Health Organization Classification of Tumours of Female Genital Organs, Fifth Edition [5].

Immunohistochemistry for WT1

Immunohistochemical staining for WT1 was performed using the peroxidase-antiperoxidase method. Commercial immunohistochemistry kits were obtained from PathnSitu (Secunderabad, India), and staining was carried out according to the manufacturer’s instructions using a rabbit monoclonal WT1 antibody (clone EP122).

Four-micrometer-thick sections mounted on poly-L-lysine-coated slides were deparaffinized in xylene and rehydrated through graded alcohols. Endogenous peroxidase activity was blocked using 3% hydrogen peroxide. Heat-induced epitope retrieval was performed in a microwave oven using Tris-ethylenediaminetetraacetic acid (EDTA) buffer. The sections were incubated with the primary antibody at room temperature in a humidified chamber, followed by incubation with a secondary antibody. Visualization was achieved using diaminobenzidine as the chromogen. The sections were counterstained with hematoxylin, dehydrated, cleared, and mounted with DPX.

The WT1 antibody used was a ready-to-use preparation, and incubation with the primary antibody and secondary antibody was carried out for 30 minutes each at room temperature in a humidified chamber.

Known WT1-positive serous ovarian carcinoma tissue was used as a positive control. Negative controls were prepared by omitting the primary antibody.

WT1 immunostaining was independently evaluated by two pathologists. Both observers were blinded to the histological subtype and FIGO stage at the time of scoring. In cases of discrepant interpretation, the slides were reviewed jointly, and a consensus score was assigned. Formal statistical assessment of interobserver reproducibility was not performed.

Evaluation of WT1 immunostaining 

WT1 immunoreactivity was assessed based on nuclear staining of tumor cells, with staining graded according to the proportion of tumor cells showing nuclear positivity, as described by Goldstein and Uzieblo (Table 1) [13].

**Table 1:** Grading system for WT1 immunohistochemical expression adapted from Goldstein et al. [14]

WT1 Staining Grade	Percentage of Tumor Cells Showing Nuclear Positivity	Interpretation
0	<5%	Focal staining
1+	5-25%	Weak positivity
2+	26-50%	Moderate positivity
3+	51-75%	Strong positivity
4+	>75%	Diffuse strong positivity

For statistical analysis, tumors showing focal nuclear staining (<5%), corresponding to the first category of the grading system, were considered WT1-negative.

Statistical analysis

Statistical analysis was performed using IBM SPSS Statistics version 29 (IBM Corp., Armonk, NY). Associations between categorical variables were assessed using Pearson’s chi-square test or Fisher’s exact test, as appropriate based on expected cell counts. WT1 expression was analyzed in relation to histological subtype, tumor category, and FIGO stage. A p-value < 0.05 was considered statistically significant.

## Results

A total of 300 histopathologically confirmed epithelial ovarian tumors were included in the present study. The clinicopathological characteristics of the tumors and the pattern of WT1 immunohistochemical findings are summarized in the following tables. All cases were analyzed with respect to age distribution, histological subtype, clinical presentation, laterality, tumor size, FIGO stage, and WT1 expression.

Of the 300 epithelial ovarian tumors studied, 240 cases (80%) were malignant, while 60 cases (20%) were borderline tumors, indicating that malignant tumors constituted the majority of cases (Table 2).

**Table 2:** Distribution of surface epithelial ovarian tumors (n = 300)

Diagnosis	Number of Cases	Percentage
Borderline	60	20%
Malignant	240	80%
Total	300	100%

The age distribution of patients showed that epithelial ovarian tumors were most frequently observed in the fourth to sixth decades of life. Malignant tumors were predominantly seen in patients aged 41-60 years, whereas borderline tumors were relatively more common in younger age groups (Table 3). A statistically significant association existed between age group and tumor behavior.

**Table 3:** Age-wise distribution of borderline and malignant epithelial ovarian tumors Fisher's exact test, p < 0.001 (statistically significant)

Age (Years)	Borderline (n)	Percent	Malignant (n)	Percentage
Up to 20	5	1.6%	-	0%
21-30	5	1.6%	10	3.3%
31-40	10	3.3%	35	11.6%
41-50	30	10%	130	43.3%
51-60	10	3.3%	35	11.6%
61-70	-	0%	25	8.3%
>70	-	-	5	1.6%
Total	60	20%	240	80%

The most common clinical presentation among patients with malignant ovarian tumors was an abdominal mass, followed by abdominal or back pain, ascites, weight loss, and urinary symptoms. Borderline tumors were more likely to present as an abdominal mass with fewer associated systemic symptoms (Table 4). Clinical presentation differed significantly between borderline and malignant tumors.

**Table 4:** Clinical presentation of epithelial ovarian tumors Fisher's exact test, p < 0.001 (statistically significant)

Clinical Presentation	Borderline (n)	Malignant (n)
Mass per abdomen	20	165
Associated pain (abdomen/back)	10	100
Amenorrhea	-	10
Postmenopausal bleeding	-	85
Urinary symptoms	-	145
Loss of weight	5	205
Ascites	-	170

With respect to laterality, the majority of ovarian tumors were unilateral, with left-sided ovarian involvement being more frequent than right-sided involvement. Bilateral tumors were observed in a smaller proportion of cases (Table 5).

**Table 5:** Laterality of epithelial ovarian tumors in the study population

Side of the Ovary Involved	Number of Cases	Percentage
Right	100	33%
Left	155	52%
Bilateral	45	15%
Total	300	100%

Tumor size at presentation varied considerably. Most tumors measured greater than 10 cm in maximum dimension, with those measuring 11-20 cm forming the largest group, followed by tumors measuring 21-30 cm. Very large tumors measuring 31 cm or more were uncommon (Table 6).

**Table 6:** Size distribution of epithelial ovarian tumors

Size (cm)	Number of Cases	Percentage
3-10	85	28.3%
11-20	110	36.6%
21-30	100	33.3%
≥31	5	1.6%
Total	300	100%

Solid and cystic consistency was the most common pattern observed in both borderline and malignant tumors. Solid and solid-cystic tumors were more frequently observed among malignant cases, with purely solid tumors occurring exclusively in malignant tumors, while cystic tumors constituted a smaller proportion of both tumor groups (Table 7). A statistically significant association was observed between tumor consistency and tumor behavior (chi-square test, p < 0.001).

**Table 7:** Tumor consistency and tumor behavior Pearson chi-square test, p < 0.001 (statistically significant)

Consistency	Borderline (n)	Malignant (n)	Total (n)	Percentage
Cystic	15	20	35	11.60%
Solid and cystic	45	180	225	75%
Solid	0	40	40	13.30%
Total	60	240	300	100%

Serous tumors constituted the most common histological subtype overall and were predominantly malignant, whereas mucinous tumors were more frequently observed among borderline tumors. Seromucinous tumors showed an equal distribution between borderline and malignant categories, while poorly differentiated adenocarcinomas were observed exclusively among malignant tumors (Table 8). A statistically significant association was observed between histological subtype and tumor behavior, indicating that serous histology was predominantly associated with malignant tumors, while mucinous histology was more commonly associated with borderline tumors (Table 9).

**Table 8:** Distribution of various types of surface epithelial ovarian tumors according to histological type Fisher's exact test, p < 0.001 (statistically significant)

Histological Type	Borderline (n, %)	Malignant (n, %)	Total (n, %)
Serous	15 (5%)	190 (63.3%)	205 (68.3%)
Mucinous	35 (11.6%)	30 (10%)	65 (21.6%)
Seromucinous	10 (3.3%)	10 (3.3%)	20 (6.6%)
Poorly differentiated adenocarcinoma	0	10 (3.3%)	10 (3.3%)
Total	60 (20%)	240 (80%)	300 (100%)

**Table 9:** Distribution of surface epithelial ovarian tumors by histological diagnosis

Type of Surface Epithelial Tumor	Number of Cases	Percentage
Borderline serous tumor	15	5%
Serous cystadenocarcinoma	190	63.30%
Borderline mucinous tumor	35	11.60%
Mucinous cystadenocarcinoma	30	10%
Borderline seromucinous tumor	10	3.30%
Seromucinous adenocarcinoma	10	3.30%
Poorly differentiated adenocarcinoma	10	3.30%
Total	300	100%

Among serous ovarian tumors, serous cystadenocarcinoma constituted the majority of cases (92.7%), while borderline serous tumors accounted for 7.3% of cases (Table 10).

**Table 10:** Distribution of serous ovarian tumors

Type	Number of Cases	Percentage
Borderline serous tumor	15	7%
Serous cystadenocarcinoma	190	92.70%
Total	205	100.00%

Early-stage disease (FIGO stages I and II) was observed in 60.5% of serous cystadenocarcinoma cases, whereas 39.5% presented with advanced-stage disease (FIGO stages III and IV) (Table 11).

**Table 11:** FIGO staging of serous cystadenocarcinomas FIGO, International Federation of Gynecology and Obstetrics

FIGO Stage	Number of Cases	Percentage
Stages I and II	115	61%
Stages III and IV	75	39.50%
Total	190	100.00%

WT1 positivity was predominantly observed in serous tumors, with 185 of 205 serous tumors showing WT1 expression. All poorly differentiated adenocarcinomas demonstrated WT1 positivity, whereas mucinous and seromucinous tumors were uniformly WT1-negative (Table 12). However, on statistical analysis, no significant association was observed between WT1 expression and tumor category (borderline versus malignant) (Fisher’s exact test, p = 1.000).

**Table 12:** WT1 positive expression in various epithelial ovarian tumors Fisher's exact test; p = 1.000; statistically not significant WT1, Wilms’ tumor 1

Histological Type	Borderline (WT1+ / Total)	Malignant (WT1+ / Total)	Total (WT1+ / Total)
Serous	5 / 15	180 / 190	185 / 205
Mucinous	0 / 35	0 / 30	0 / 65
Seromucinous	0 / 10	0 / 10	0 / 20
Poorly differentiated adenocarcinoma	-	10 / 10	10 / 10
Total	5 / 60	190 / 240	195 / 300

Lower WT1 expression scores were more frequently observed in early-stage (FIGO stages I and II) serous cystadenocarcinomas, whereas higher WT1 expression scores (+3 and +4) were predominantly seen in advanced-stage tumors (FIGO stages III and IV) (Table 13). A statistically significant association was observed between WT1 expression level and FIGO stage in serous cystadenocarcinomas, with higher WT1 scores being associated with advanced-stage disease.

**Table 13:** WT1 score vs. FIGO stage in serous cystadenocarcinomas Fisher's exact test, p < 0.001 (statistically significant) FIGO, International Federation of Gynecology and Obstetrics; WT1, Wilms’ tumor 1

WT1 Score	FIGO Stages I and II	FIGO Stages III and IV
0	10	0
+1	55	0
+2	40	5
+3	10	25
+4	0	45
Total	115	75

WT1 expression was predominantly observed in serous carcinomas, with the majority of serous carcinomas demonstrating WT1 positivity, whereas all mucinous carcinomas were WT1-negative (Table 14). Pearson chi-square test demonstrated a statistically significant association between WT1 expression and histological type, indicating that WT1 positivity was strongly associated with serous carcinoma and absent in mucinous carcinoma (p < 0.001).

**Table 14:** Pearson chi-square test showing the association between WT1 expression and histological type Pearson chi-square test, p < 0.001 (statistically significant) WT1, Wilms’ tumor 1

Histological Type	WT1 Positive	WT1 Negative
Serous carcinoma	180	10
Mucinous carcinoma	0	30

## Discussion

The present study evaluated the immunohistochemical expression of WT1 in epithelial ovarian tumors and correlated its expression with histological subtype and FIGO stage. A total of 300 cases, including borderline and malignant epithelial ovarian tumors, were analyzed to assess the diagnostic relevance of WT1 and to compare the findings with previously published studies.

In the present study, malignant epithelial ovarian tumors constituted 80% of cases, while borderline tumors accounted for 20%. This distribution is comparable to hospital-based studies reported by Pilli et al. and Gupta et al., which also demonstrated a predominance of malignant tumors in tertiary care centers [14,15]. Such findings differ from population-based cancer registries, where borderline tumors are less frequently represented, reflecting referral bias and the advanced nature of cases managed at specialized institutions [1,4].

The peak age incidence of epithelial ovarian tumors in the present study was observed in the 41-60-year age group. This finding is consistent with epidemiological observations reported by Reid et al. and Kurman and Shih, who noted that epithelial ovarian carcinomas predominantly affect middle-aged and older women [16,17]. Borderline tumors were more frequently encountered in younger patients, a pattern also reported by Seidman and Kurman, Silverberg, and Köbel et al., supporting the relatively indolent biological behavior of borderline ovarian tumors [18-20].

Clinically, malignant tumors in the present study commonly presented with abdominal mass, ascites, weight loss, and urinary symptoms. These nonspecific symptoms are well documented and have been emphasized by Lheureux et al. as major contributors to delayed diagnosis in ovarian cancer [21]. Borderline tumors, in contrast, demonstrated fewer systemic manifestations, consistent with their limited invasive potential.

Histologically, serous tumors constituted the most common epithelial ovarian tumor subtype in the present study, followed by mucinous tumors. This distribution closely mirrors findings from multiple studies, including the WHO classification, Kurman and Shih, and Köbel et al., which consistently identify serous carcinoma as the predominant epithelial ovarian malignancy [3,17,18]. The proportion of mucinous tumors observed in the present study is also comparable to that reported in similar institutional series.

With respect to FIGO staging, a substantial proportion of serous cystadenocarcinomas in the present study presented at advanced stages. This observation is consistent with reports by Reid et al. and Lheureux et al., who highlighted the aggressive biological behavior and early peritoneal dissemination of high-grade serous carcinomas [16,21]. FIGO staging remains the standard framework for assessing disease extent and prognostic stratification in ovarian carcinoma [22].

WT1 immunohistochemical expression showed a distinct and consistent pattern across histological subtypes in the present study. Strong nuclear WT1 positivity was observed in the majority of serous carcinomas, while mucinous and seromucinous tumors were uniformly negative. These findings are in close agreement with studies by Köbel et al., Goldstein and Uzieblo, and Soslow et al., all of whom demonstrated high WT1 positivity in serous ovarian carcinomas and absence of expression in mucinous tumors [11-13,23]. Similar diagnostic utility of WT1 in epithelial ovarian cancer has also been demonstrated by Hylander et al. [9].

WT1 immunohistochemical expression in epithelial ovarian tumors has been well documented by Shimizu et al., who demonstrated preferential WT1 expression in serous ovarian carcinomas with the absence of staining in most non-serous subtypes [24]. The findings of the present study closely parallel these observations, further validating the role of WT1 as a marker of serous differentiation.

WT1 was originally identified as a tumor suppressor gene implicated in WT1 and has since been shown to play a role in the development and differentiation of several malignancies, including ovarian cancer [25]. This biological role supports its continued relevance as a diagnostic marker in ovarian tumor pathology.

In the present study, poorly differentiated adenocarcinomas also demonstrated WT1 positivity. Similar observations were reported by Soslow et al., who emphasized that WT1 expression may be retained in poorly differentiated tumors of serous lineage even when morphological features are ambiguous [23]. This supports the concept that WT1 reflects tumor lineage rather than degree of differentiation.

Analysis of WT1 expression in relation to FIGO stage revealed that higher WT1 scores were more frequently observed in advanced-stage serous carcinomas. Comparable associations have been reported by Hylander et al. and Goldstein and Uzieblo, who demonstrated that WT1 expression is closely linked to tumor biology and disease burden in epithelial ovarian cancer [9,13]. However, other studies have reported variable or absent correlations between WT1 expression and survival outcomes, indicating that while WT1 is a robust diagnostic marker, its prognostic significance remains uncertain [26].

The statistically significant association between WT1 expression and histological subtype observed in the present study further supports findings from previous studies demonstrating lineage-specific WT1 expression in epithelial ovarian tumors [11-13,23,24]. The complete absence of WT1 expression in mucinous carcinomas highlights the value of WT1 as part of a routine immunohistochemical panel in the classification of ovarian tumors.

The strengths of the present study include systematic histopathological evaluation, standardized immunohistochemical assessment of WT1 expression, and correlation with histological subtype and FIGO stage. Limitations include the relatively small sample size and the lack of long-term follow-up data to assess prognostic outcomes. Despite these limitations, the findings of the present study are largely concordant with published literature and contribute further evidence supporting the diagnostic relevance of WT1 in epithelial ovarian tumors.

Clinical implications

Accurate histopathological classification of epithelial ovarian tumors is essential for appropriate clinical management and prognostication. The findings of the present study highlight the practical value of WT1 immunohistochemistry as a diagnostic adjunct in routine surgical pathology practice, particularly in cases with overlapping morphological features or poor differentiation.

WT1 is especially useful in distinguishing serous carcinomas from mucinous and seromucinous tumors and in identifying serous lineage in poorly differentiated carcinomas. Incorporation of WT1 into standard immunohistochemical panels, along with other lineage-specific markers, may improve diagnostic accuracy, reduce interobserver variability, and facilitate appropriate therapeutic decision-making.

At present, WT1 should be regarded primarily as a diagnostic marker rather than a standalone prognostic indicator, as variability exists regarding its association with clinical outcomes across studies.

Future directions

Future studies with larger sample sizes and multicentric cohorts are required to further validate the diagnostic and potential prognostic role of WT1 expression in epithelial ovarian tumors. Inclusion of long-term follow-up data, such as overall survival and progression-free survival, would help clarify the relationship between WT1 expression and clinical outcomes.

Integration of WT1 immunohistochemical findings with molecular and genetic profiling may provide deeper insights into the biological role of WT1 in ovarian tumorigenesis and progression. Further research exploring WT1-associated molecular pathways could also contribute to the identification of novel prognostic markers or therapeutic targets in serous ovarian carcinoma.

## Conclusions

The present study evaluated the immunohistochemical expression of WT1 in epithelial ovarian tumors and correlated its expression with histological subtype and FIGO stage. WT1 demonstrated a distinct lineage-specific expression pattern, with strong nuclear positivity predominantly observed in serous ovarian carcinomas and in poorly differentiated adenocarcinomas of serous lineage, while mucinous and seromucinous tumors consistently lacked WT1 expression. A statistically significant association was observed between WT1 expression and histological subtype, reinforcing the diagnostic utility of WT1 in distinguishing serous carcinomas from non-serous epithelial ovarian tumors. Higher WT1 expression scores were more frequently observed in advanced-stage serous carcinomas, suggesting a possible association with tumor biology, although its independent prognostic significance remains uncertain.

Overall, the findings support the role of WT1 as a reliable immunohistochemical marker in the routine evaluation and classification of epithelial ovarian tumors.
